# Mechanism of Single-Cycle THz Pulse Generation and X-ray Emission: Water-Flow Irradiated by Two Ultra-Short Laser Pulses

**DOI:** 10.3390/nano13182505

**Published:** 2023-09-05

**Authors:** Hsin-Hui Huang, Saulius Juodkazis, Eugene G. Gamaly, Vladimir T. Tikhonchuk, Koji Hatanaka

**Affiliations:** 1 Optical Sciences Centre, School of Science, Swinburne University of Technology, Hawthorn, VIC 3122, Australia; 2 ARC Training Centre in Surface Engineering for Advanced Materials (SEAM), School of Science, Swinburne University of Technology, Hawthorn, VIC 3122, Australia; 3 Research Center for Applied Sciences, Academia Sinica, Taipei 115, Taiwan; 4 WRH Program International Research Frontiers Initiative (IRFI), Tokyo Institute of Technology, Nagatsuta-cho, Midori-ku, Yokohama 226-8503, Japan; 5 Laser Physics Centre, Research School of Physics and Engineering, The Australian National University, Canberra, ACT 0200, Australia; 6 Centre Lasers Intenses et Applications, University of Bordeaux, 351 Cours de la Liberation, 33405 Talence, France; 7 Extreme Light Infrastructure ERIC, ELI Beamlines Facility, Za Radnicic 835, 25241 Dolní Břežany, Czech Republic; 8 Research Administration Office, Organization for Research Strategy and Development, Okayama University, Okayama 700-8530, Japan; 9 Center for Optical Research and Education (CORE), Utsunomiya University, Tochigi 321-8585, Japan

**Keywords:** water ablation, THz emission, dipole radiation, coherent transient radiation, Bremsstrahlung emission

## Abstract

The interaction of two subsequent ultra-short sub-milli-Joule laser pulses with a thin water flow results in an emission of a strong single-cycle THz pulse associated with enhanced soft X-ray emission. In this paper, a chain of processes produced in this interaction is analyzed and compared with other THz generation studies. It is demonstrated that the enhanced THz and X-ray emissions are produced by an energetic electron beam accelerated in the interaction of a main laser pulse with liquid water ejected from the surface by the pre-pulse. This scheme thus provides an efficient laser energy conversion in a THz pulse, avoiding laser self-focusing and filamentation in air.

## 1. Introduction

Interaction between intense femtosecond laser at near-IR (photon energy ∼1 eV) and matter can produce emission in a broad range from X-ray at several-keV to THz wave at meV [1,2,3,4,5]. THz radiation can be used to control light and matter interactions [6,7,8,9,10,11,12] and it is very appealing due to non-ionizing nature of the interaction. Phonons in solid-state materials, absorption at the manifold of molecular vibrations in gas/liquid/solid phases as well as next generation of high-speed communication such as 6G technology [13,14] are all using this sub-mm wavelength spectral domain. THz radiation can originate from sources such as gases [15,16,17,18,19,20,21], atomic clusters [22,23,24,25], liquids [26,27], and solids [28,29] as well as photoconductive antennas [30,31], photo-mixers [32], difference frequency generation in a nonlinear all-dielectric nanoantennas [33], and laser-induced plasmas [34,35]. Compact and intense sources of X-rays and THz are in demand due to their expanding applications in monitoring, metrology, sensing, security [36,37,38,39,40] and imaging/spectroscopy [41,42,43]. Especially for THz, sub-wavelength sources are highly attractive for the possibility they offer to control the intensity, phase, polarization, and angular momentum of the beam [44,45]. However, when ultra-short laser pulses are used for the generation of THz, filamentation, and color mixing [46] makes such sources less practical due to extended dimensions.

The observations from space and time-resolved experiments were reported on the generation of a single-cycle THz wave from a well-timed and positioned two fs-laser pulses irradiating on a micro-thin water flow in air [47]. The polarization of THz pulse can be changed from linear to circular by adjusting the position of focusing of the second, stronger laser main-pulse. When the experiment was conducted under the same focusing conditions but without a water flow, it resulted in the generation of a weaker THz emission with a polarization aligned with the linear polarization of the main-pulse [48]. The conclusion drawn was that the polarization of the THz emission is defined by the asymmetric density profile of the shockwave front in the air, induced by the pre-pulse [48]. These THz sources are sub-wavelengths at the THz spectral range.

**Figure 1:**
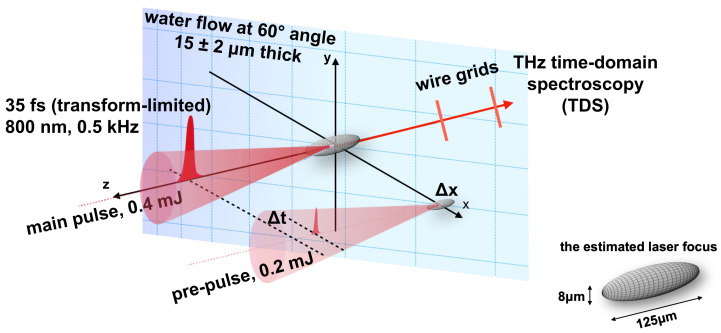
Two-pulse irradiation on the micro-thin water flow. Time domain spectroscopy (TDS) was used to detect THz radiation from the irradiation zone. See Figure 2 for the detailed geometry of the light interaction with the water jet.

In this study, we analyze the interaction conditions of two consecutive laser pulses with a thin water flow (Figure 1) and propose a scenario for the generation of THz radiation [47], combined with enhanced X-ray emission observed experimentally [49]. The main feature of the proposed scenario is water droplets ejected from the water surface prepared by the pre-pulse irradiation is the origin of the X-ray and THz wave; this is due to the energetic electrons interacting with the smooth water surface. Furthermore, a shockwave produced in water by the pre-pulse alters the curvature of the water surface, thereby influencing the polarization of THz emission. Considering the energy deposition and the geometry of the experiment should guide specific experiments on discrimination between the above-mentioned scenario and the hypothesis involving electron acceleration in the plasma within the laser wakefield near the water surface [47].

## 2. Experimental: Samples and Methods

The detailed experimental setup for double-pulse irradiation conditions on micro-thin water flow is as described elsewhere [47,48] and is briefly outlined below; see schematics of the experiment in Figure 1.

**Figure 2:**
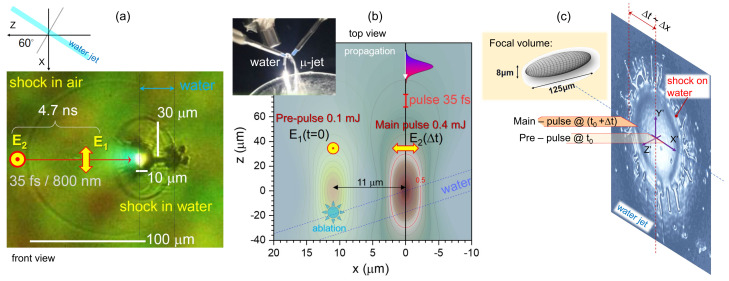
Two-pulse irradiation of the water micro-sheet at different orientations. (**a**) A backlit time-resolved shadowgraphy (yz-plane or a front-view) by white light continuum (WLC) at Δt=4.7 ns after a 0.1 mJ pre-pulse (the main-pulse of 0.4 mJ is not irradiated when this shadowgraph image was taken). (**b**) Top-view (xz-plane) positions of laterally separated pre- and main-pulses. The Inset photo shows water micro-sheet formed from colliding water jets. (**c**) The timing of the second (main) pulse is linked to the surface perturbation arrival at the point of irradiation from the ablation caused by the first pulse. Schematics of experiment of two-pulse irradiation of water flow at 60° angle of incidence; note, a tilted coordinate frame $x^{\prime }y^{\prime }z^{\prime }$ is shown in (**c**) to make water surface as a $x^{\prime }y^{\prime }$-plane as used for discussion of surface shockwaves. The image used for the schematics is a rendered slide glass ablation with one fs-laser pulse. It is used to illustrate the generic phenomenon of ablation crater formation, side walls, and microflows/droplets. Inset shows the focal volume of laser pulses in air.

A pulsed femtosecond laser (λ=800 nm, $t_{p}=35$ fs, transform-limited, 1 kHz, Mantis, Legend Elite HE USP, Coherent, Inc.) is used, and the output pulses were split into the pre-pulse ($E_{1}$, vertically-polarized, 0.2 mJ/pulse) and the main pulse ($E_{2}$, horizontally-polarized, 0.4 mJ/pulse) with fine spatio-temporal control over the two pulses. An off-axis parabolic mirror (OAPM, 1-inch diameter, focal length *f* = 50.8 mm, 47-097, Edmund Optics) is used to focus the pre-pulse and the main pulse onto a micro-thin water sheet (∼17 μm). The angle of incidence of laser at the water flow is 60° to maximize p-polarization coupling with the water target, resulting in higher X-ray [50] and THz emission [51] as shown in Figure 2a. These emissions are enhanced by the double pulse excitation condition with the main pulse irradiating after the pre-pulse with a temporal delay, Δt. The highest THz enhancement is achieved with an additional spatial offset of the pre-pulse at ($\Delta x_{1}$, $\Delta y_{1}$, $\Delta t_{1}$) = (11 μm, 0 μm, 0 ns) and the main pulse at ($\Delta x_{2}$, $\Delta y_{2}$, $\Delta t_{2}$) = (0 μm, 0 μm, 4.7 ns) [47], the geometry and focusing conditions are as shown in Figure 2b. The spatial offsets along the *x*- and *y*-axes, $\Delta x_{1}$ and $\Delta y_{1}$, are achieved with a set of automatically controlled piezo-transducer mirrors (POLARIS-K2S2P, KPZ101, Thorlabs).

After the OAPM with the effective numerical aperture (NA) at 0.125 [47], the laser is focused at the water surface to a spot area of $S_{f}=4.3\times 10^{-7}$ cm${}^{2}$ with 1.22λ/NA≈7.4μm spot diameter and 120μm focal depth. The corresponding fluences for the pre-pulse and the main pulse are F=460 and 920 J/cm${}^{2}$, respectively. This is hundreds of times higher than the ablation threshold of ∼2 J/cm${}^{2}$ in the wide-bandgap $E_{g}>6$ eV materials [52,53]. At this intensity range of 1.3 and 2.6 $\times \;10^{16}$ W/cm${}^{2}$, both pulses ionize air in the tunneling regime, and 1–2 electrons are stripped off from oxygen and nitrogen. This corresponds to the under-critical plasma density $n_{e}<n_{cr}$ for λ=800 nm, where the critical density $n_{cr}=\omega^{2}\varepsilon_{0}m_{e}/e^{2}=1.74\times 10^{21}$ cm${}^{-3}$ with the cyclic frequency $\omega =2\pi c/\lambda =2.36\times 10^{15}$ [1/s], $\varepsilon_{0}$ is the permittivity of the free space, $m_{e}$ is the electron mass and *e* is the elementary charge.

An objective lens (M Plan Apo 10×, MITUTOYO) and a CMOS camera (Blackfly S USB3, FLIR Systems, Inc.) were used to capture the time-resolved shadowgraphy as shown in Figure 2a. It shows the shockwave generated from the pre-pulse irradiating the thin water sheet. The main pulse is redirected to induce a white light continuum (∼1 ps, 580 ± 30 nm selected with color filters, as a strobe light) illuminating along the *x*-axis [48]. Figure 2a,c show the shockwave on the yz-plane and the surface condition of the water after pre-pulse irradiation and ejection of micro-droplets. Light localization within the depth-of-focus 100–120μm for the air breakdown was observed experimentally by shadowgraphy. Strong focusing and a pulse power close to the critical self-focusing threshold of $P_{cr}=5.4$ GW/pulse (at λ∼1μm) in air [54] are the reasons for a well-defined energy deposition and localization. Visualization of the air breakdown under the experimental conditions showed that the axial extent is ∼120–150μm, which is close to the depth-of-focus defined by $2z_{R}$. The Rayleigh length is $z_{R}=\pi nw_{0}^{2}/\lambda \approx 60\;\mu$m with $w_{0}=0.61\lambda /NA$ defining the waist of the beam for the focusing, which corresponds to the numerical aperture of NA=0.125. The laser pulse has a spatial extent of $c\times t_{p}\approx 13\;\mu$m, and it traverses the focal volume of $2z_{R}$ length ionizing air (via tunneling ionization); the air is not strongly absorbing/reflecting due to $n_{e}<n_{cr}$. It takes ∼10 fs or just a few optical cycles of 2.7 fs (at 800 nm) to ionize the air and create a hot plasma cylinder along the focal region $2z_{R}\approx 120\;\mu$m-long.

The pressure *P* generated by a shockwave in air under breakdown in similar fs-laser exposure conditions (360 fs, 1030 nm, 0.5 PW/cm${}^{2}$) is found closely following the dependence $P=\left(\gamma -1\right)\frac{\rho }{\rho_{0}}\epsilon$, where ϵ is the internal energy per unit volume, $\gamma =C_{p}/C_{v}=1.4$ is the specific capacity ratio at constant pressure to that at constant volume of the diatomic gas, ρ is the density of air, $\rho_{0}=1.204\times 10^{-3}$ g/cm${}^{3}$ is the unperturbed/atmospheric air density [54]. Maximum compression of air up to ∼3 times by the shockwave is observed using shadowgraphy [54]. It is revealing that the shockwave compressed air front expands to ∼11μm distance, which corresponds to the most efficient THz generation [47] (Figure 2).

Transmission THz time-domain spectroscopy (THz-TDS) and its polarization measurements are performed with the conventional electro-optic sampling method with a set of wire-grids, a 〈110〉-oriented ZnTe crystal (1-mm thick, Nippon Mining & Metals Co., Ltd.), a balanced photo-diode (Model2307, New Focus), and a lock-in amplifier (SR830, Stanford Research System) [47,55,56,57,58]. All the experiments were carried out under normal conditions: in air under atmospheric pressure (1 bar), room temperature (296 K), and 40–50% humidity.

## 3. Results: Light-Matter Interaction Conditions

### 3.1. Scenarios of X-ray and THz Radiation

The scenario of laser interaction with the water sheet is as follows: First, the laser pre-pulse focused at the water surface deposes its energy and produces two effects: (i) generates a strong shockwave in the water and (ii) ejects droplets of liquid water and water vapors out of the surface (Figure 2). The second laser pulse arrives at the water surface with a delay of a few nanoseconds, and its focal position is shifted by about 11μm with respect to the pre-pulse (Figure 2b). The efficient THz and X-ray emissions occur if the main laser pulse is focused at the position of the shockwave front at the water surface and crosses the water droplets on its way (Figure 2c). Then, the interaction of the main pulse with water droplets results in the generation of a beam of energetic electrons that propagate in the laser beam direction and interact with the curved water surface at the shockwave front. The interaction of the electron beam with water molecules results in a strong Bremsstrahlung emission, and, at the same time, the electron beam crossing the air-water interface produces a dipole electromagnetic emission in the THz domain. The angle of incidence of electrons at the water surface defines the polarization of THz pulse. This process can also be interpreted as a coherent transient radiation of the electron beam crossing the water surface, as shown below.

Let us start from estimates and present conjectures of THz and X-ray generation at the tight focusing conditions considering that the light-matter interaction is confined within the geometrical focus, which has been established by direct observation [47] (see, Figure 2a). Self-focusing and filamentation are not present. Energy deposition at the front side of the water sheet by pre-pulse triggers shockwaves in the air and on the water surface as shown in Section 3.2. The conjectures and required parameters are estimated in the following analysis of experimental results. In such an approach, we can estimate separately predictions of the energy deposition, THz, and X-ray emission along with the polarization and temporal properties of the radiation.

### 3.2. Shockwave in Air and Water

**Figure 3:**
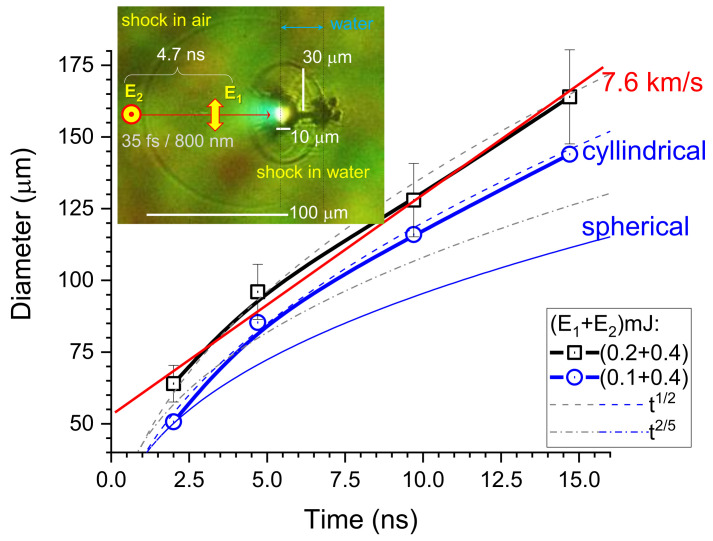
Temporal evolution of the diameter of a shockwave front as a function of delay time, Δt, after the energy $E_{1}$ deposition of the pre-pulse. The inset shows a shadowgraph at the delay of 4.7 ns; the pre-pulse energy is 0.1 mJ (same as Figure 2a). The back-light illumination through a 17-μm-thick water sheet is made by the white light continuum (WLC) produced by the main pulse $E_{2}$. The apparent thickness of the 17-μm water flow is twice larger in projection, i.e., 34 μm (front view) due to the 60° angle of incidence onto the water sheet. The time evolution of the shockwave radius according to the Sedov–Taylor model in the case of spherical (i=3) and cylindrical (i=2) explosion follows $R\propto t^{2/(i+2)}$; see the dashed and dash-dotted lines (Equation (1)). Slope of 7.6 μm/ns is shown (red line as an eye guide); the error bars are 10%.

**Sedov–von Neumann–Taylor blast wave**. As shown in Figure 3, temporal evolution of the shockwave front is clearly observed in the shadowgraphy. A dynamic explosion triggered by the pre-pulse is seen, and a strong shockwave emission is observed. It is informative to present solution for the point-like energy deposition [59], which is more appropriate to the shockwave generated on water surface. The radius, *R*, of the shockwave is increasing in time *t* as [59,60]:

(1) $$R\left(t\right)={\left(\frac{E_{p}}{K\left(\gamma \right)\rho_{0}}\right)}^{1/(2+i)}t^{2/(2+i)},$$

where $E_{p}$ is the instantaneous deposition of energy in space and time, K(γ)=(0.6−0.8) is the constant depending on the adiabatic coefficient $\gamma =C_{p}/C_{v}$ (γ=1.4 for air), $\rho_{0}$ is the mass density of unperturbed medium (air), and *i* is the dimensionality coefficient which is i=2 for spherical and i=3 for cylindrical explosions, respectively. As shown in Figure 3, the comparison of the experimental result with the models based on spherical (i=2) and cylindrical (i=3) explosion indicates that the shockwave expansion in air is in between of the two limits. A cylindrical explosion is a better fit for the experiment. The diameters plotted in Figure 3 correspond to a circle, which fits the transverse cross section of the shockwave affected region.

The maximum volume occupied by a shockwave in air at pressure of $p_{0}=1$ bar can be estimated from the absorbed pulse energy $E_{p}$ divided by the spherical shocked volume with radius $r_{sh}=\sqrt[{3}]{\frac{3E_{p}}{4\pi p_{0}}}$. It is $r_{sh}=980\;\mu$m for $E_{2}=0.4$ mJ (corresponds to a fully absorbed energy of the second pulse). At this distance, the shockwave transforms into a pressure wave and travels at the speed of sound under normal conditions.

The density jump across the shockwave front is given by [60]:

(2) $$\frac{\rho }{\rho_{0}}=\frac{\left(\gamma +1\right)M^{2}}{\left(\gamma -1\right)M^{2}+2},$$

where $M=v_{sh}/v_{s}$ is the Mach number defined by the ratio of $v_{sh}$ shockwave to $v_{s}$ sound velocities ($v_{s}=0.343$ km/s in air at normal conditions). The shockwave with speed $v_{sh}=7.6$ km/s is generated with a cumulative action of two pulses $E_{1}+E_{2}=0.6$ mJ, which corresponds to a strong explosion with Mach number M=22.2. The density compression of $\rho /\rho_{0}=5.9$ (Equation (2)) occurs across the shockwave front. The pressure enhancement at the shockwave front is [60]:

(3) $$\frac{P}{P_{0}}=\frac{2\gamma M^{2}-\left(\gamma -1\right)}{\gamma +1}$$

and reaches $\frac{P}{P_{0}}=572.6$. With these estimates for pressure and density, one would expect a refractive index of air n>2.2 at the THz spectral band (see Appendix A).

**Figure 4:**
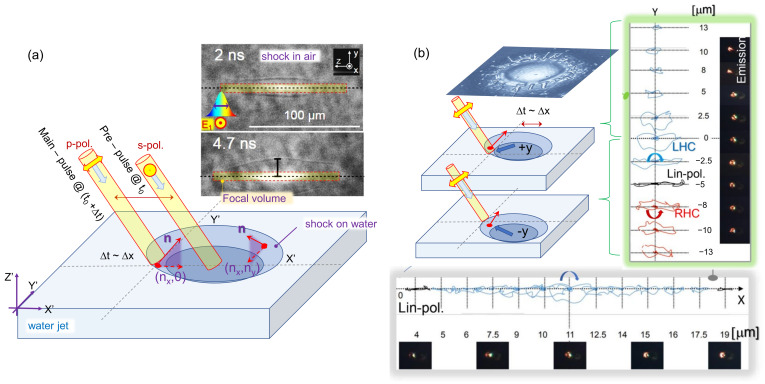
Polarization of THz radiation depends on the (x,y) position of the pre-pulse with respect to the main pulse (0, 0). (**a**) Schematics of interaction on the surface of thin water flow at the moment of arrival of the main-pulse at a time delay Δt after pre-pulse; the plane of incidence in xz-plane. The **n** is the normal to the front of shockwave with different projections (*n_x_*, *n_y_*) dependent on the location. Ejected droplets and shockwave in pre-surface air induced by pre-pulse are clearly discernible in shadowgraphy (Figure 2a) but not shown here for simpler schematics. The diameter of laser pulse on the *xy*-plane is comparable with the perturbation of shocked water. Shadowgraphy insets show the focal volume and air densification. (**b**) Left hand circular and right hand circular (LHC and RHC) polarizations of THz emission at different half-planes in respect to the positive and negative *y* position values for pre-pulse focus [47]. Projections of electric vector traces shown for reflected THz radiation (adopted from ref. [47]). The top-inset is a rendered SEM image of a glass ablation site by a single fs-laser pulse (same as in Figure 2c). Thumbnail images of time-integrated optical emission are shown side-by-side with THz polarization traces.

The shockwave is also excited in the water sheet (Figure 3). It is better visualized with a low energy pre-pulse $E_{1}=0.1$ mJ since a single line-like modification throughout the entire water sheet is observed rather than multi-filaments produced at larger pulse energies. The shocked region inside water forms a cylinder of 35 μm radius at 15 ns ($v_{sh}=2.3$ km/s) corresponding to M=1.6 for sound speed in water $v_{s}=1.48$ km/s. At M<2, the explosion cannot be considered as strong. The most prominent feature of water irradiation with a pair of sub-mJ-pulses is a strong shockwave launched into surrounding air and along the surface of water sheet (see. Figure 4).

### 3.3. X-ray Emission

Let us start with the measured values of the energetic efficiency of emission in the X-ray domain. According to the experimental results presented in the paper by K. Hatanaka et al. [61], the energy conversion of laser to X-rays is $\eta_{X}=1.3\times 10^{-8}$, and Figure 4 in Ref. [61] shows that photons have an exponential distribution in energy with the effective temperature of $T_{X}=2$ keV. It is known that an exponential spectrum of Bremsstrahlung photons corresponds to the exponential spectrum of electrons with approximately the same temperature, $T_{e}≃T_{X}=2$ keV. This temperature agrees also with the energy of electron oscillations in the focus of the main laser pulse.

The efficiency of Bremsstrahlung emission of electron beam entering a target of thickness larger than the electron stopping range is calculated by M. Lamoureux and P. Charles [62]. For electrons having Maxwellian distribution with a temperature $T_{e}$, the energy conversion efficiency is:

(4) $$\eta_{X-e}=1.8\times 10^{-6}ZT_{e},$$

where *Z* is the effective ion charge and $T_{e}$ is given in keV. For a water target Z=4.75 (the average charge in the mixture is calculated as $\sum Z_{i}^{2}n_{i}/\sum Z_{i}n_{i}$ where $Z_{i}$ is the charge of species *i* and $n_{i}$ its relative concentration). For $T_{e}=2$ keV, the emission efficiency is $\eta_{X-e}=1.7\times 10^{-5}$. Since $\eta_{X}=\eta_{X-e}\eta_{e}$, we can estimate the efficiency of laser energy conversion into electrons, $\eta_{e}=0.76\times 10^{-3}$ in the experiment. That corresponds to the total energy of hot electrons is $E_{e}=\eta_{e}E_{\mathrm{las}}=0.3\;\mu$J. Moreover, since the average energy of electrons is ${\bar{\epsilon }}_{e}=1.5T_{e}=3$ keV, the total number of hot electrons of $N_{e}=E_{e}/{\bar{\epsilon }}_{e}=1\times 10^{9}$. This corresponds to the electric charge $Q_{e}=eN_{e}=64$ pC. Therefore, only a small fraction of laser energy is transferred to these hot electrons. The remaining laser energy is transferred to low energy electrons, which produce water heating but not relevant to radiations. We show now that this small fraction of accelerated electrons can be also responsible for the THz emission.

### 3.4. THz Emission

According to the paper by H.H. Huang et al. [47], the laser energy conversion into THz is $\eta_{THz}=1.6\times 10^{-5}$, which corresponds to the conversion efficiency in terms of the number of photons $\left(6-7\right)\times 10^{-3}$. For the main-pulse laser energy of $E_{\mathrm{las}}=0.4$ mJ, the energy of THz pulse is $E_{THz}=6.4$ nJ. Assuming the pulse duration $t_{THz}=1$ ps, the emission power is $P_{THz}=6.4$ kW; THz detector was 70 cm away from the fs-pulse interaction zone with water flow.

Two emission mechanisms can be considered: coherent transition radiation or dipole emission. In the former case, the electrons have to be created in air or water vapors just before the water surface and emit radiation while entering the water. The electron pulse duration is equal to the THz pulse duration $t_{THz}=1$ ps multiplied by the electron average velocity, which is about $v_{e}≃25\;\mu$m/ps (corresponding to energy of 3 keV), the length of electron bunch is $l_{e}=v_{e}t_{THz}≃25\;\mu$m. It is about one-fifth of the laser pulse Rayleigh length, thus suggesting that electron acceleration takes place by the central part of the laser pulse near the water surface.

**Transition radiation**. Let us consider first the transition radiation. It is produced by a charge traversing the boundary between two media with dielectric permittivities $\varepsilon_{1}$ and $\varepsilon_{2}$. For a non-relativistic electron crossing the interface air-water ($\varepsilon_{1}≃1$ and $\varepsilon_{2}≃10\gg 1$), the emission energy per unit frequency range is [63]:
  (5) $$\frac{dE_{\mathrm{trans}}}{d\nu }=\frac{8}{3}\frac{r_{c}}{c}E_{e}.$$

Here, $r_{c}$ is the electron classical radius, $E_{e}$ is the electron energy and dν is the spectral width of emission. For $d\nu ≃1/t_{THz}≃1$ THz, the conversion efficiency is $E_{\mathrm{trans}}/E_{e}=\eta_{\mathrm{trans}}≃2.5\times 10^{-11}$. However, since the characteristic emission wavelength is longer than the electron beam length, the emission is coherent, and the total emitted energy is the energy emitted by a single electron multiplied by the square of number of electrons $N_{e}$. The product $N_{e}E_{e}$ gives the total energy of electron beam, consequently, the fraction of electron energy converted into radiation is:

(6) $$\eta_{THz\;CTR}=\frac{8r_{c}}{3c}\;\frac{N_{e}}{t_{THz}}≃0.025.$$

Recalling that the laser-to-electron conversion efficiency estimated from the X-ray emission in Section 3.3 is $\eta_{e}=0.76\times 10^{-3}$, we find the efficiency of laser energy conversion into THz emission $\eta_{THz-e\;CTR}\eta_{e}≃1.9\times 10^{-5}$. This value is in good agreement with the experimentally measured efficiency. Thus, the coherent transient emission of laser-accelerated electrons is a valid candidate for the THz source.

**Dipole emission**. Another way to describe the THz emission is to consider the electron beam created in the laser focus as an electric current abruptly stopped at the water surface. Indeed, the length of electron beam $l_{e}=25\;\mu$m is larger than the stopping length of 3 keV electron in water, which is ∼1μm . The power of dipole emission is given by the Larmor formula (see, for example, textbook [64]):

(7) $$P_{\mathrm{dipole}}=\frac{\pi }{12}\zeta_{0}J_{e}^{2}\frac{l_{e}^{2}}{\lambda_{THz}^{2}},$$

where $J_{e}=Q_{e}/t_{THz}=160$ A is the electric current in the dipole, $\zeta_{0}=377\;\Omega$ is the vacuum impedance and $\lambda_{THz}=300\;\mu$m is the THz wavelength. This formula gives the dipole emission power of $P_{\mathrm{dipole}}=17.5$ kW, which is just three times larger than the experimental value. Thus, the THz source can also be identified as a transient dipole.

There is, in fact, no contradiction between these two explanations of the THz emission. Coherent transient emission and transient dipole emission are two complementary visions of the same process. The principal point is that the radiation is created by a time-dependent asymmetric charge separation: immobile ions in the zone of electron acceleration and electron current abruptly stopped at the water surface.

The electric current and consequently radiated electric field are lying in the plane defined by the direction of propagation of the electron beam (that is, the main laser pulse) and the normal to the water surface. Consequently, if the plane of incidence of the main laser pulse at the water surface is the same as the pre-pulse, that is, the main pulse is incident at $y_{b}=0$ in Figure 2c, and it is p-polarized, the THz emission is polarized in the same plane. By contrast, for the point of incidence of the main pulse $y_{b}\neq 0$, the normal to the water surface is out of the plane of incidence, and the radiated electric field has both components $E_{x}$ and $E_{y}$, with *y* component having the same sign as the focusing point coordinate $y_{b}$. This geometry results in an elliptical polarization of THz emission. The rotation of polarization is related to the curvature of the bump on the shockwave front and the temporal profile of the electron bunch. This conjecture needs further in-depth analysis experimentally and theoretically.

## 4. Discussion

We are coming now to the last step of our analysis: how the electrons are accelerated, what is the role of laser pre-pulse, and why the time delay of a few ns is needed. Without pre-pulse, the optimum configuration for efficient electron acceleration corresponds to the focusing laser close to the water surface. So, there is a sufficient length for electron acceleration in the air, and the conducting surface is very close to the acceleration zone, which permits a partial transformation of the electrostatic field of the electron bunch into the radiation.

However, the acceleration of electrons in the focus of a non-relativistic laser pulse is inefficient because the laser electric field is perpendicular to the acceleration direction, and magnetic field contribution is of the second order. For this reason, the X-ray and THz emissions produced by a single pulse are inefficient.

The presence of the matter ablated or ejected from a solid surface dramatically changes the interaction conditions for the second (main) pulse. In particular, the laser interaction with liquid droplets of a micrometric size increases the laser plasma coupling and facilitates electron acceleration.

The effect of double pulse liquid surface irradiation with two pulses was studied in Ref. [65]. The experiments were conducted in a vacuum with the main pulse of 1 mJ and 55 fs irradiating a surface of liquid gallium. The main pulse intensity of $4\times 10^{16}$ W/cm${}^{2}$ was very similar to the present experiment.

It was observed that the X-ray emission in the range of 5–50 keV is strongly enhanced if the main pulse was preceded by a pre-pulse, with energy 10–100 times smaller than the main pulse and arriving a few ns before. The efficiency of conversion of laser energy into X-rays was on the order of $10^{-7}$, ten times larger than in the present experiment. This enhancement of conversion efficiency is consistent with the fact that electrons were interacting with gallium atoms having charge Z=31, and the electron average energy was 2–3 times larger than in the present experiment.

**Figure 5:**
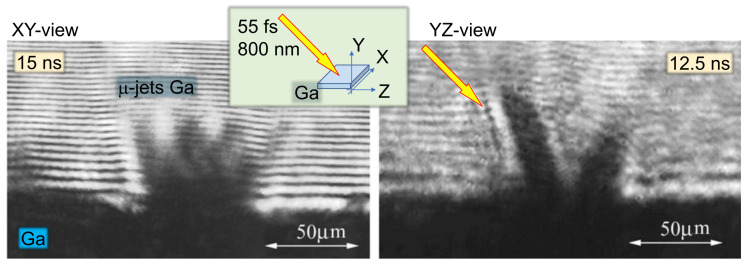
Images of plasma plume at the surface of melted Ga target obtained at a time delay of 12.5 ns in two perpendicular directions in the target plane. The yellow arrow shows the direction of pre-pulse beam. Images are adapted from the paper by Uryupina et al. [65].

The origin of an efficient electron acceleration in the two-pulse configuration has been identified with the optical shadowgraphy. Figure 5 shows images of a plasma plume at the surface of a melted Ga target obtained at a time delay of 12.5 ns in two perpendicular directions in the target plane. It was found that this delay is optimum for the X-ray production with the main pulse.

In difference from a homogeneous plasma plume produced routinely at the surface of solid targets, here the plume is structured; it contains 2–3 dense flows surrounded by vapors. It is very likely that these flows correspond to gallium in a liquid phase. The possibility of liquid droplet formation in the case of laser energy deposition in the range of 5–50 J/cm${}^{2}$ has been confirmed in the dedicated hydrodynamic simulations with a wide-range equation of state accounting for the phase transitions.

Since the irradiation conditions are rather similar, it is reasonable to suppose that the similar ejection of flows and droplets in a liquid phase had happened in the present experiment [47]. However, since the water density is 6 times smaller than the density of gallium, the velocity of ejecta is higher, and, consequently, the optimum conditions for the electron acceleration are achieved with shorter delays of 4–5 ns. Numerical modeling of light field localization and enhancement on sub-wavelength and wavelength-sized water droplets was demonstrated in X-ray generation by a pair of pulses [66]. It contributes to ionization and electron production along the main pulse.

Efficient acceleration of electrons in the presence of liquid ejecta has been confirmed with the kinetic particle-in-cell simulations presented in the same paper [65]. The jets were modeled as cylinders of a diameter of 2 laser wavelengths and a length of 10 wavelengths. The number and energy of accelerated electrons increased significantly in the presence of flows. The effect of electron acceleration is explained by the presence of strong longitudinal electric fields that accelerate electrons along the jet surface. Although the parameters of numerical simulation do not correspond exactly to the experimental conditions, they demonstrate well the physical processes leading to efficient electron acceleration.

Next, let us discuss possible ways to enhance X-ray and THz emission from the water target. Comparing ∼1 kbar pressures generated by pre-pulse with the water bulk modulus K≈20 kbar, we conclude that the shockwave initially is compressible, but at the distances about d∼11μm it becomes incompressible; K≡−VdP/dV, where *P* and *V* are the pressure and the volume in water. So, the bulge at the surface at the time of main pulse arrival is of the same order as the shockwave width. The size of the laser beam waist of 3–4μm can be considered as an estimate for the shockwave width and the height of the bulge. So, the size of the electron beam (generated via the linear momentum deposition) is comparable with the water surface curvature. Larger pre-pulse energy, as well as spatially separated irradiation positions for pre-pulses could be used to produce steeper surface profiles as well as nano-/micro-droplets generation, which interact with the main pulse. Electrons oscillate in the laser pulse with the energy of the order of the ponderomotive energy $W_{p}=I_{p}/n_{cr}c$. This corresponds to the energy of accelerated electrons of 2–3 keV. Thanks to the presence of water droplets, the number of accelerated electrons is much larger than in the fact of air alone [67,68,69] (Section 3.3 and Section 4).

In addition to the ponderomotive energy, electrons are gaining momentum in the direction of laser propagation. However, for non-relativistic laser intensities, the drift velocity is much smaller than the quiver electron velocity, and it can be neglected. Indeed, $v_{D}∼0.25a_{0}^{2}c$, that is about $1\times 10^{6}$ m/s = 1 μm/ps. It is about 10 times smaller than the quiver electron velocity. Knowing the length of the electron beam and its average velocity, we estimate the duration of interaction 100μm/1μm/ps = 100 ps. The accelerated electrons interact with the water surface. The stopping range of a 3 keV electron in water is about 1 μm, and the corresponding lifetime is 1 ps, which defines the time of electromagnetic emission. So, electrons cannot penetrate through the water layer, and emission comes from the front surface only, which is consistent with direct optical observations by shadowgraphy [47].

## 5. Conclusions and Outlook

Joint analysis of THz and X-ray emissions from the double pulse interaction with a water layer allows us to reduce significantly the range of parameters and constrains the possible explanations. The energy and spectrum of X-ray emission indicate that it is produced by hot electrons with a temperature of about 2 keV and total energy of 0.2μJ. The same electrons are at the origin of THz emission, which can be considered as a dipole emission of an electron current entering water or as a coherent transient emission of a short electron bunch accelerated by the main laser pulse. The direction and polarization of THz emission are defined by the orientation of the dipole with respect to the normal to the water surface.

Efficient acceleration of electrons near the water surface in the presence of pre-pulse is explained by the ejection of sub-micrometric jets or droplets from the water surface in the focus of laser pulse. These opaque, conducting structures transform an electromagnetic laser field into an electrostatic field, which efficiently accelerates electrons.

Short THz pulses of several optical cycles emitted from sub-wavelength (for THz) volumes can be used to create THz emitters to probe materials at a small scale. Due to its small size, coherent THz emitters have a larger intensity. The very same THz emitter also produces hard X-rays, which can augment material characterization capabilities. As this study shows, the light-matter interaction can be characterized using THz and X-ray radiations produced from the interaction micro-volumes. It can provide a better understanding of processing conditions for fs-laser machining at intensities entering the 1–10 PW/cm${}^{2}$ range.

## Data Availability

Data underlying the results presented in this paper are not publicly available at this time but can be obtained from the authors upon reasonable requests.

## Appendix A. Air Properties

**Refractive index of air at high pressure**. Refractive index of air at pressures up to 5 bar have been directly measured using the THz time-domain spectroscopy in the wide spectral range [70]. The refractive index *n* was found closely following the Hauf–Grigull law dependence $n\left(\rho \right)-1=\frac{3}{2}\frac{r_{n}MP}{RT}$, where ρ is the density, *r* is the refractivity of gas, *M* is its molar mass, *P* is the pressure, *R* is the ideal gas constant, *T* is the absolute temperature of gas. At constant temperature (e.g., across the shockwave front), the refractive index depends on the gas pressure and molar refractivity $r_{n}=\frac{M}{\rho }\frac{n^{2}-1}{n^{2}+2}$, which defines the polarizability $\alpha^{{}^{\prime }}=3r_{n}/\left(4\pi N_{A}\right)$; here $N_{A}$ is the Avogadro number. The refractive index follows the linear dependence vs. pressure as determined from direct measurements for the tested gases; for N${}_{2}$ (air) $n=1+2.39\times 10^{-3}P$ [70]. At a very high pressures of ∼500 atm encountered at the shockwave front of a strong explosion, the refractive index would reach n≈2.2.

**Air properties under normal conditions.** The velocity of air molecules at normal conditions is $v_{rms}=\sqrt{3k_{B}T/m}$; 480 m/s for O${}_{2}$ and 510 m/s for N${}_{2}$. During a laser pulse of $t_{p}=40$ fs, N${}_{2}$ molecules only move 0.2 Å, which is smaller than a molecular size (diameter) a≈1.5 Å. The free path of molecules is $l_{f}=1/\left(\sqrt{2}\pi n_{0}\sigma \right)=k_{B}T/\left(\sqrt{2}\pi p\sigma \right)$, where $n_{0}=2.69\times 10^{19}$ cm${}^{-3}$ is the number density of molecules, $\sigma =a^{2}$ is the cross-section of molecules defined by their size *a*, p=1 bar is the pressure, $k_{B}$ is the Boltzmann constant, and *T* is the absolute temperature. The value of $l_{f}$ is ≈60 nm in air.

Mass density of air $\rho_{air}=1.29\times 10^{-3}$ g/cm${}^{-3}$ (at 0 °C), which has molecular number density $n_{0}=\rho_{air}N_{A}/M_{air}$. The average molar mass of air as 79% of N_2_ and 21% of O_2_ is $M_{air}=0.79\times 28+0.21\times 32=28.84$ g. The atomic mass ratio (average) of air $M_{air}/N_{A}$ to electron mass: $\frac{m_{av}}{m_{e}}\equiv \frac{M/N_{A}}{m_{e}}=5.25\times 10^{4}$, where $N_{A}$ is the Avogadro number. Hence, the electron-to-ion (average) mass ratio $m_{e}/m_{av}=3.8\times 10^{-5}$.

Dielectric strength of air with the index of refraction $\tilde{n}\approx 1$ at the normal conditions (room temperature and pressure) is $E_{b}^{air}=3$ MV/m. The corresponding light intensity $I_{a}=c\varepsilon_{0}{\left[E_{b}^{air}\right]}^{2}/2\approx 119.4$ TW/cm${}^{2}$. The air breakdown occurs at smaller intensities for $t_{p}=1$ ps: ∼20 TW/cm${}^{2}$ and is dependent on the pulse duration as $\propto 1/\sqrt{t_{p}}$. The main reason of a lower dielectric breakdown threshold is due to contribution of avalanche ionization in generation of electrons and ions. For a very short pulse $t_{p}<1$ ps, the avalanche ionization becomes less significant and the breakdown threshold has a constant value. The pulse intensity of $I_{p}=13$ PW/cm${}^{2}$ (pre-pulse in this study) corresponds to the electric field strength of $E=3.1\times 10^{11}$ V/m. This is close to the atomic electric field strength, which is for the hydrogen atom with a electron-proton separation of $r_{H}=53$ pm is $E_{H}=\frac{1}{4\pi \varepsilon_{0}}\frac{e}{r_{H}^{2}}=5.1\times 10^{11}$ V/m.
